# First national record of *Microhylahmongorum* Hoang, Nguyen, Phan, Pham, Ninh, Wang, Jiang, Ziegler and Nguyen, 2022 (Anura, Microhylidae, *Microhyla*) in China

**DOI:** 10.3897/BDJ.11.e103580

**Published:** 2023-04-10

**Authors:** Yun-He Wu, Zhong-Bin Yu, Chen-Qi Lu, Kasyoka Kilunda Felista, Shao-bing Hou, Jie-Qiong Jin, Jin-Min Chen, Dong-Ru Zhang, Zhi-Yong Yuan, Jing Che

**Affiliations:** 1 Southeast Asia Biodiversity Research Institute, Chinese Academy of Sciences, 05282, Yezin, Nay Pyi Taw, Myanmar Southeast Asia Biodiversity Research Institute, Chinese Academy of Sciences, 05282 Yezin, Nay Pyi Taw Myanmar; 2 State Key Laboratory of Genetic Resources and Evolution & Yunnan key laboratory of biodiversity and ecological conservation of Gaoligong Mountain, Kunming Institute of Zoology, Chinese Academy of Sciences, 650223, Kunming, Yunnan, China State Key Laboratory of Genetic Resources and Evolution & Yunnan key laboratory of biodiversity and ecological conservation of Gaoligong Mountain, Kunming Institute of Zoology, Chinese Academy of Sciences, 650223 Kunming, Yunnan China; 3 Kunming College of Life Science, University of the Chinese Academy of Sciences, 650204, Kunming, Yunnan, China Kunming College of Life Science, University of the Chinese Academy of Sciences, 650204 Kunming, Yunnan China; 4 Key Laboratory of Conserving Wildlife with Small Populations in Yunnan, Southwest Forestry University, 650224, Kunming, Yunnan, China Key Laboratory of Conserving Wildlife with Small Populations in Yunnan, Southwest Forestry University, 650224 Kunming, Yunnan China

**Keywords:** Amphibia, *Microhylahmongorum*, new record, China

## Abstract

**Background:**

To date, 10 species of the genus *Microhyla* have been recorded in China, of which six were distributed in Yunnan Province. *Microhylahmongorum* Hoang, Nguyen, Phan, Pham, Ninh, Wang, Jiang, Ziegler, and Nguyen, 2022 was also speculated to be distributed in Xishuangbana, Yunnan Province, China. However, there is no evidence of documentation of *M.hmongorum*.

**New information:**

We report the first country record of *Microhylahmongorum*, based on specimens collected from Yunnan border region. Morphologically, the specimen was consistent with the original descriptions of *M.hmongorum*. Phylogenetically, the sequences of the specimens from China clustered with the sequence of type specimens of *M.hmongorum* from Vietnam, with uncorrected pairwise distances of 0.9% at the 16S gene fragment analysed. Therefore, we report *M.hmongorum* as a new record species in China.

## Introduction

*Microhyla* Tschudi, 1838, a genus of the family Microhylidae, is widely distributed throughout India and Sri Lanka eastwards to the Ryukyu Archipelago of Japan and southwards to Indonesia and presently includes 51 recognised species (Frost 2023). To date, 10 species have been recorded in China, namely *M.beilunensis* Zhang, Fei, Ye, Wang, Wang and Jiang, 2018, *M.berdmorei* (Blyth 1856), *M.butleri* Boulenger, 1900, *M.dabieshanensis* Zhang, Chen and Zhang, 2022, *M.fanjingshanensis* Li, Zhang, Xu, Lv and Jiang, 2019, *M.fissipes* Boulenger, 1884, *M.heymonsi* Vogt, 1911, *M.mixture* Liu and Hu, 1966, *M.mukhlesuri* Hasan, Islam, Kuramoto, Kurabayashi and Sumida, 2014 and *M.pulchra* (Hallowell, 1861) (AmphibiaChina 2023). Of these 51 species, six occur in Yunnan, *M.berdmorei*, *M.butleri*, *M.fissipes*, *M.heymonsi*, *M.mukhlesuri* and *M.pulchra* (Yuan et al. 2022). Hoang et al. (2022) studied the distribution pattern of the *M.heymonsi* group with descriptions of two new species (*M.hmongorum* and *M.xodangorum*) from Vietnam, which postulated the distribution of *M.hmongorum* in Xishuangbana, Yunnan Province, China. However, there is no evidence of documentation of *M.hmongorum* in China. To date, this species was known only from the type locality in Vietnam.

Yunnan Province borders Vietnam, Laos and Myanmar. Recently, several cryptic and novel amphibians’ species have been described in the border region (e.g. Yang and Huang (2019), Yuan et al. (2019), Chen et al. (2020), Liu et al. (2021), Wu et al. (2021), Zhang et al. (2022)). These findings imply that the amphibian diversity in the border region may still be diverse and largely underestimated. During our fieldworks in Yunnan, China, we collected some specimens of rice frogs, which can be assigned to the genus *M.heymonsi* complex. Detailed morphological comparisons and molecular analysis indicated that these specimens should be categorised as *M.hmongorum*; thus, we herein describe the new records in details.

## Materials and methods

**Figure 1.**
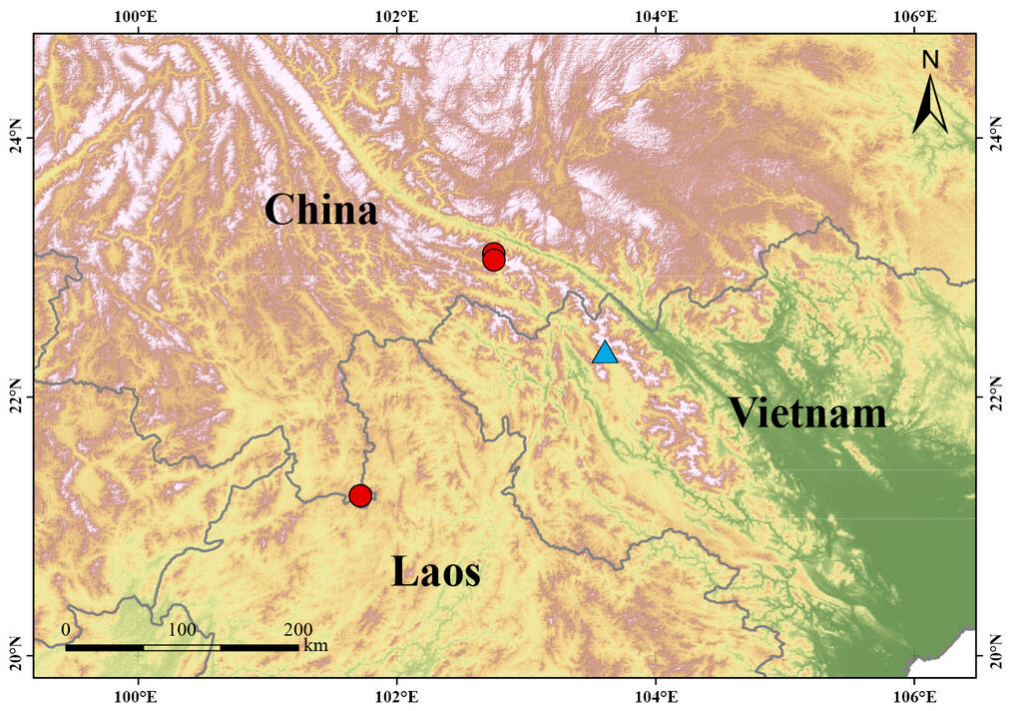
Map showing the new record in China (red circle) and the type locality of *M.hmongorum* (blue triangle) in Vietnam.

Field surveys were conducted in Xishuangbanna and Yuanyang, Yunnan Province, China in April 2016 and September 2017 (Fig. 1). These specimens were euthanised and fixed in 10% formalin and then transferred to 70% ethanol for permanent storage. Liver tissue samples were preserved in absolute ethyl alcohol for molecular analysis. The tissue sample was deposited in the Herpetological Museum of Kunming Institute of Zoology (KIZ), Chinese Academy of Sciences (CAS).

Total genomic DNA was extracted from liver tissues using the standard phenol-chloroform extraction protocol (Sambrook et al. 1989). The mitochondrial gene 16S ribosomal RNA gene (16S rRNA) was amplified and sequenced from six specimens using the primer pairs (5’–3’) 16S rRNA-F (CGCCTGTTTAYCAAAAACAT) and 16S rRNA-R (CCGGTYTGAACTCAGATCAYGT) (Kocher et al. 1989). PCR amplifications were performed in a 25 μl reaction volume with the following procedure: initial denaturing step at 95°C for 4 min, 35 cycles of denaturing at 94°C for 40 s, annealing at 55°C for 1 min and extending at 72°C for 1 min and a final extension at 72°C for 10 min. The amplified PCR product was purified using Qiagen PCR purification kit and then sequences in both directions were obtained from an ABI 3100 automated sequencer. New sequences were deposited in GenBank under accession numbers (the GenBank accession numbers are available in Suppl. material 1). Newly-obtained sequences were first assembled and edited using AutoSeqMan (Sun 2018).

Phylogenetic relationships amongst *M.heymonsi* complex were inferred using Maximum Likelihood (ML) and Bayesian Inference (BI) to reconstruct phylogenetic relationships. Homologous sequences of *M.heymonsi* complex and outgroup (*M.marmorata*) were obtained from GenBank (Suppl. material 1). New sequences incorporated with homologous data retrieved from GenBank were aligned using MUSCLE 3.8 (Edgar 2004) and then inspected by eye for accuracy and trimmed to minimise missing characters in MEGA6 (Tamura et al. 2013). Both BI and ML analyses were executed in the CIPRES web server (Miller et al. 2010). The GTR+I+G model was selected as the best substitution model by jModelTest 2.1.4 (Darriba et al. 2012). The BI analyses used Metropolis Coupled Markov Chain Monte Carlo (MCMC) with three heated chains and one cold chain for 10 million generations and sampled every 1,000 generations, with the first 25% of samples discarded as burn-in. Maximum Likelihood analyses were performed using RAxML-HPC BlackBox 8.2.10 (Stamatakis 2014). The analyses used the proportion of invariable sites estimated from the data and 1,000 bootstrap pseudoreplicates under the GTR+G model. Pairwise divergences (uncorrected p-distance) between species on 16S dataset were calculated using MEGA6 (Tamura et al. 2013).

Measurements were recorded to the nearest 0.1 mm with digital calipers by Zhong-Bin Yu following Fei et al. (2009). Measurements included: SVL (Snout-vent length); HL (Head length); HW (Head width); SL (Snout length); INS (Internasal space); IOS (Interorbital space); NED (nasal to eye distance); UEW (Upper eyelid width); ED (Eye diameter); TD (tympanum diameter); LAL (lower arm length); LAHL (Length of lower arm and hand); HAL (Hand length); LAD (Diameter of lower arm); FEM (Femoral length); TL (Tibia length); FTL (Foot length).

## Data resources

**Figure 2.**
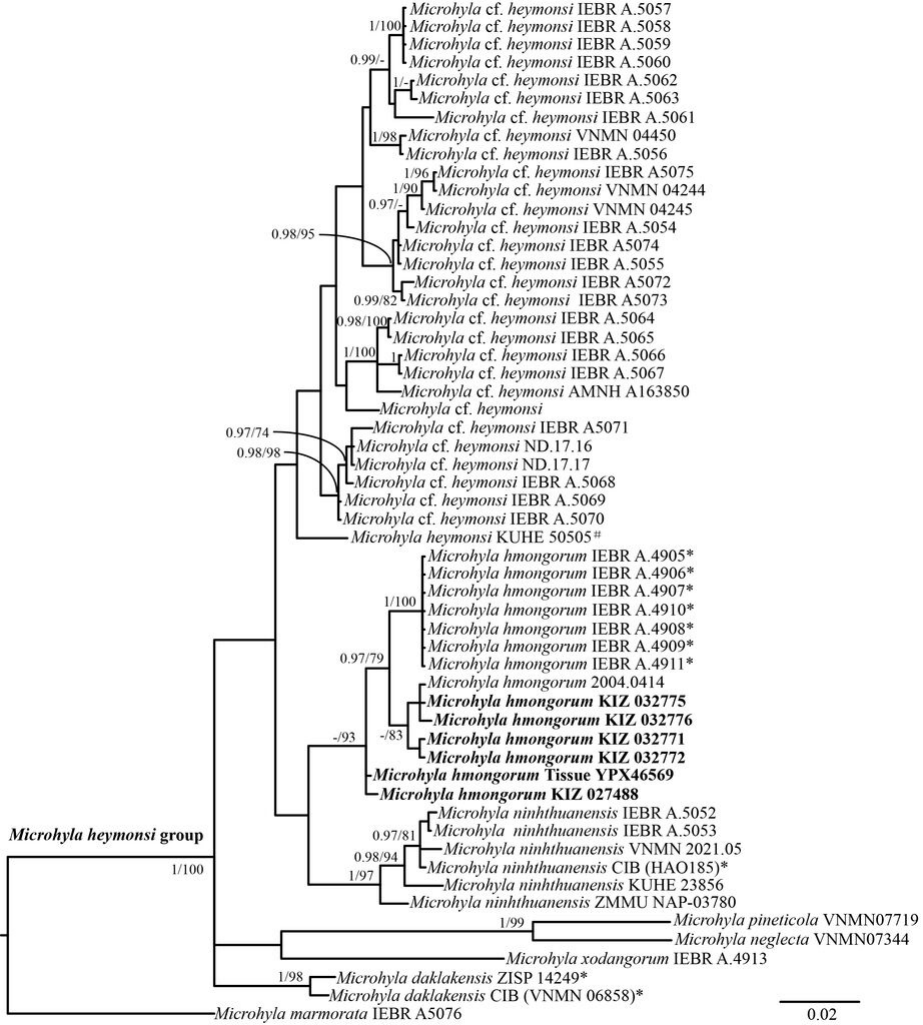
Phylogenetic tree of *M.heymonsi* complex, based on Bayesian Inference of a fragment of the mitochondrial 16S gene. Nodal support values with Bayesian posterior probability (BPP) > 0.95 / ML inferences (BS) > 70 are performed near the respective nodes. A “-” Bayesian posterior probability < 0.95 and bootstrap support < 70. Bayesian posterior probability (BPP) < 0.95 / ML inferences (BS) < 70 are not shown.

The aligned 16S dataset contained a total of 1153 nucleotide base pairs (bp) in length, with 269 variable positions and 176 parsimony informative sites (including outgroups). The BI and ML analyses showed consistent topology (Fig. 2). The results indicated that the monophyly of the *Microhylaheymonsi* group was strongly supported and in agreement with results of Hoang et al. (2022). These specimens collected from Mengla and Yuanyang, Yunnan, China, clustered with the specimens (including the type specimens) of *M.hmongorum* from Vietnam (Fig. 2). Genetic divergence between the specimens from China and the type specimens of *M.hmongorum* was only 0.9% (Table 1). It is comparable to interspecific genetic divergence (uncorrected *p*-distance) between the new sample obtained from Yunnan, China and the other species of *Microhylaheymonsi* group varied from 4.0% (versus M.cf.heymonsi) to 11.1% (versus *M.neglecta*) (Table 1). Morphologically, the specimen from Yunnan Province shows a similar appearance to the original description of *M.hmongorum*. Therefore, we considered the Yunnan, China population to be conspecific with *M.hmongorum*.

Table 1

## Taxon treatments

### Microhyla hmongorum

Hoang, Nguyen, Phan, Pham, Ninh, Wang, Jiang, Ziegler and Nguyen, 2022

58FE596A-4C48-52FE-BECF-0CC825AE391F

#### Materials

- **Type status:** Other material. **Occurrence:** catalogNumber: KIZ 027488; individualCount: 1; sex: male; lifeStage: adult; **Taxon:** acceptedNameUsage: *Microhylahmongorum*; class: Amphibia; order: Anura; family: Microhylidae; genus: Microhyla; specificEpithet: *hmongorum*; **Location:** country: China; countryCode: CHN; stateProvince: Yunnan; county: Yuanyang; locality: Panzhihua; verbatimElevation: 1375 m; verbatimLatitude: 23°3′14.89″; verbatimLongitude: 102°44′58.09″; **Record Level:** basisOfRecord: preserved specimen

#### Description

**Figure 3.**
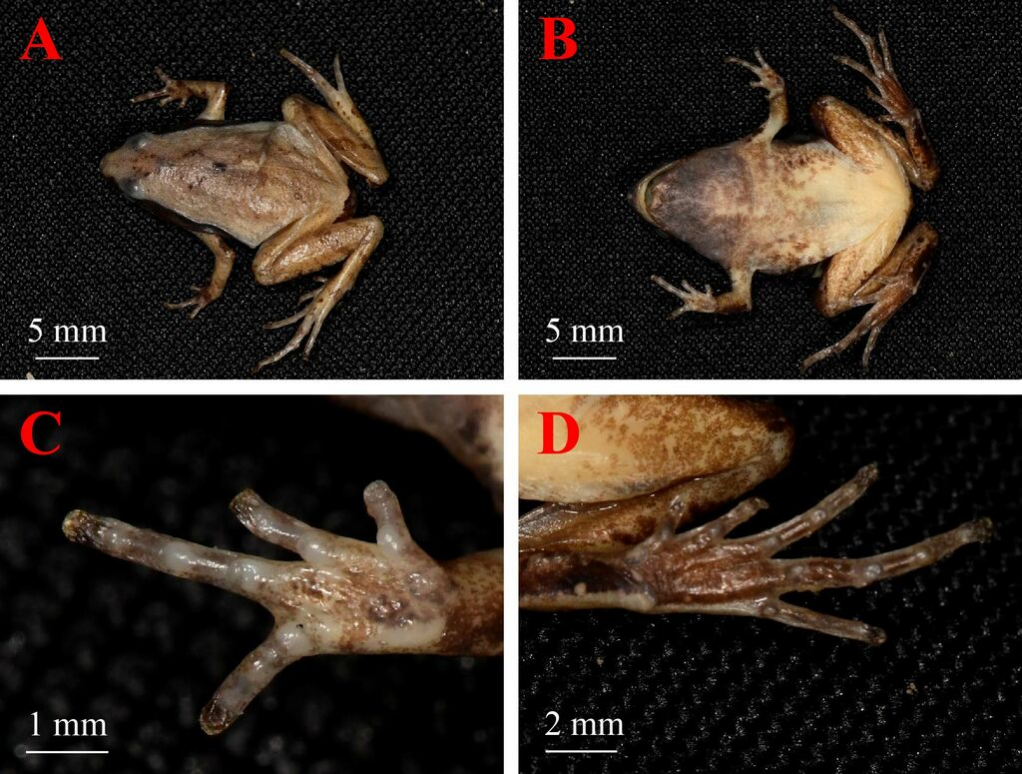
Male of *M.hmongorum* (KIZ 027488) in preservative. **A** Dorsal view; **B.** Ventral view; **C** Ventral view of figure; **D** Ventral view of foot.

Morphmetrics of the speciemen are provided in see Suppl. material 2. Small size frog, body triangle, adult male with SVL 19.9 mm; head length (HL 6.2 mm, 32.1% of SVL) slightly longer than width (HW 5.8 mm, 29.1% of SVL); snout rounded in profile, projecting beyond the lower jaw, its length (SL 2.9 mm, 14.6% of SVL) longer than horizontal diameter of eye (ED 1.9 mm, 9.5% of SVL); canthus rostralis round, loreal region vertical and slightly concave; interorbital space ﬂat, larger (IOS 2.1 mm, 10.6% of SVL) than width of upper eyelid (UEW 1.4 mm, 7.0% of SVL) and internarial distance (INS 1.9 mm, 9.5% of SVL); snout longer than eye diameter (SL/ED 152.6%); tympanum hidden; vomerine teeth absent; tongue posteriorly oval and not notched behind; supratympanic fold weak, extending from posterior corner of eye to arm insertion; male with internal single subgular vocal sac; nuptial pad absent (Fig. 3).

Forelimbs slender; lower arm length (LAL 3.4 mm, 17.1% of SVL) shorter than hand length (HAL 4.8 mm, 24.1% of SVL); relative finger lengths: I<IV<II<III; tips of all fingers slightly enlarged; no webbing between fingers; subarticular tubercles distinct, round, formula: 1, 1, 2, 2; three metacarpal tubercles, middle metacarpal tubercle oval, smaller than outer and inner metacarpal tubercle and not contacting outer or inner metacarpal tubercle (Fig. 3).

Hind-limbs long, tibia (TL 10.5 mm) about half SVL and shorter and foot (FL 52.8 mm); relative length of toes: I<II<V<III<IV; tibiotarsal articulation reaching between nostrils and eyes; heels overlapping when thighs are positioned at right angles to the body; tips of toes rounded and not swollen; rudimentary webbing between toes; subarticular tubercles distinct, round, formula 1, 1, 2, 3, 2; inner metatarsal tubercle elongated, outer metatarsal tubercle prominent, large (Fig. 3).

Dorsal skin surface relatively smooth with small tubercles; ventral surfaces of body and limbs smooth; flanks of body relatively smooth (Fig. 3).

In preservation. Dorsal surfaces of body and limbs greyish or brown, with usually a yellow hair-fine median line from snout to anus and two very small black spots on back, forming “ ()” -shape; ventral surfaces of body whitish obscured by many brown marblings; flanks and lateral side of head dark with a dark lateral stripe; ventral side of throat of adult male black; dorsal parts of limbs, fingers and toes with brown crossbars (Fig. 3).

#### Distribution

*Microhylahmongorum* was previously known in Lai Chau Province, northern Vietnam, Phongsali and Luang Prabang Provinces of Laos and Kachin of Myanmar (Hoang et al. 2022). Our study further extends the species' distribution range northwards to Mengla, Xishuangbanna and Yuanyang, Honghe, Yunnan Province, China.

#### Ecology

The species is often found in areas that are highly disturbed by human activity. The habitat of the species mainly includes paddy fields, still ponds and rain puddles. Breeding season of the species is mainly during April to September. This species is in sympatric distribution with *M.butleri*, *M.mukhlesuri* and *F.multistriata*.

#### Notes

Morphological characters of the specimen from China agreed well with the original description of Hoang et al. (2022). Based on the type locality in northern Vietnam, we suggest “Yuè Běi Jī Wā (越北姬蛙)” as its Chinese common name.

## Discussion

Yunnan Province, renowned for its diverse range of species, is located in southwest China and lies at a biological crossroads of three biodiversity hotspots; the Himalaya, mountains of southwest China and Indo-Burma (Mittermeier et al. 2004). China tops the list of amphibian diversity amongst these biodiversity hotspots (Jiang et al. 2016). In addition, 36 new amphibian species from Yunnan have been discovered and identified over the past three years (AmphibiaChina 2023). These results indicate that the region’s rich amphibian diversity is still underestimated. Our study confirms that specimens from Mengla, Xishuangbanna and Yuanyang, Honghe, Yunnan Province, China belong to *M.hmongorum*, representing the first record of this species in China. The discovery of *M.hmongorum* in this study raises the overall number of known amphibian species in China from 634 (AmphibiaChina 2023) to 635, along with the known number of *Microhyla* species from 10 to 11 (Frost 2023). Notably, this brings the number of *Microhyla* species recorded in Yunnan, China, to seven, namely *M.hmongorum*, *M.berdmorei*, *M.butleri*, *M.fissipes*, *M.heymonsi*, *M.mukhlesuri* and *M.pulchra*. Our findings further validate the underestimation of amphibian diversity in Yunnan. Furthermore, this species is currently known from two isolated areas in Yunnan: Xishuangbannan and Honghe which are separated by a straight-line distance of approximately 200 km. However, the species is likely to occur in other parts of Yunnan, China. Therefore, more samples from China should be included to conduct detailed studies on *M.heymonsi* complex to clarify the distribution range of species, especially to further confirm the distribution of *M.heymonsi* in Yunnan in the future.

Our findings further support the need to prioritise future attention to the diversity and taxonomy of amphibians in the southwest border region. Although various studies have clarified the taxonomy and geographic ranges of some species, such as *Amolopsviridimaculatus* (Zhang et al. 2021, Mahony et al. 2022), *Limnonecteslimborgi* (Huang et al. 2022) and published a series of new recorded species, new recorded genera and new species have been discovered in border region in recent years (e.g. Yuan et al. (2019), Wu et al. (2021), Zhang et al. (2022)), Amphibian diversity still remains underrated while the taxonomy of several species, such as *A.mengyangensis* (Wu et al. 2020) and *Micrylettainornata* (Liu et al. 2021) continue to be controversial. Moreover, one of the newly-recorded species we reported was found along the China-Laos border. Given its location in the same zoogeographic region, we presume that this species might also be present in northern Laos. As a result, we recommend international collaboration to strengthen fieldwork along the south-western borders paired with the integration of molecular and acoustic data in order to uncover more new species and new record species and, therefore, clarify taxonomy questions.

## Supplementary Material

XML Treatment for Microhyla hmongorum

03710621-EDD8-55E6-B269-89C3EDFDEA3B 10.3897/BDJ.11.e103580.suppl1 Supplementary material 1 Table S1 Data type Sampling information Brief description Localities, voucher ID and GenBank numbers for all samples used in this study. File: oo_832586.docx https://binary.pensoft.net/file/832586 Yun-He Wu, Zhong-Bin Yu, Chen-Qi Lu, Felista Kasyoka Kilunda, Shao-bing Hou, Jie-Qiong Jin, Jin-Min Chen, Dong-Ru Zhang, Zhi-Yong Yuan, Jing Che

077B1F60-20F7-5FC8-A4DB-07A9A63E4E71 10.3897/BDJ.11.e103580.suppl2 Supplementary material 2 Table S2 Data type Morphological data Brief description Measurement (in mm) and proportions of the *Microhylahmongorum*. File: oo_824849.docx https://binary.pensoft.net/file/824849 Yun-He Wu, Zhong-Bin Yu, Chen-Qi Lu, Felista Kasyoka Kilunda, Shao-bing Hou, Jie-Qiong Jin, Jin-Min Chen, Dong-Ru Zhang, Zhi-Yong Yuan, Jing Che

## Figures and Tables

**Table 1.** Uncorrected p-distances amongst the *Microhylaheymonsi* group (below the diagonal) and standard error estimates (above the diagonal). The ingroup mean uncorrected p-distances are shown on the diagonal.

**Species**	**1**	**2**	**3**	**4**	**5**	**6**	**7**	**8**	**9**
*M.ninhthuanensis*	**1.4**	1.0	0.9	1.0	1.3	1.5	1.2	0.8	1.5
*M.daklakensis*	5.4	**0.2**	0.9	1.0	1.3	1.5	1.3	1.0	1.6
M.cf.heymonsi	4.5	4.8	**1.7**	0.7	1.2	1.4	1.1	0.8	1.5
*M.heymonsi*	4.8	5.3	3.3	—	1.3	1.5	1.3	0.9	1.5
*M.pineticola*	9.1	8.4	7.7	8.4	—	1.1	1.3	1.3	1.6
*M.neglecta*	10.8	10.2	9.7	9.9	5.2	—	1.5	1.5	1.6
*M.xodangorum*	7.8	7.7	6.6	7.9	8.6	11.4	—	1.2	1.6
*M.hmongorum*	4.1	5.6	4.0	4.5	9.3	11.1	6.6	**0.9**	1.5
*M.marmorata*	12.4	12.1	11.6	12.5	13.0	13.2	13.2	11.3	—
