# Supplementary material for: First national record of Microhylahmongorum Hoang, Nguyen, Phan, Pham, Ninh, Wang, Jiang, Ziegler and Nguyen, 2022 (Anura, Microhylidae, Microhyla) in China
Source: Biodivers Data J. 2023 Apr 10;11:e103580. doi: 10.3897/BDJ.11.e103580 (PMC10848633; doi:10.3897/BDJ.11.e103580)
Supplement: Supplementary material 1 — Table S1 [file bdj-11-e103580-s001.docx]

Table S1. Localities, voucher ID, and GenBank numbers for all samples used in this study.

| **Species** | **Vouch ID** | **Locality** | **GenBank Accession** | **References** |
| --- | --- | --- | --- | --- |
| *Microhyla ninhthuanensis* | VNMN 2021.05 | Phuoc Binh NP, Ninh Thuan, Vietnam | MT808934 | Hoang et al. 2021 |
| *M. ninhthuanensis* | CIB (HAO185) | Phuoc Binh NP, Ninh Thuan, Vietnam | MT808935 | Hoang et al. 2021 |
| *M. ninhthuanensis* | IEBR A.5052 | Vinh Cuu, Dong Nai, Vietnam | ON723392 | Hoang et al. 2022 |
| *M. ninhthuanensis* | IEBR A.5053 | Vinh Cuu, Dong Nai, Vietnam | ON723393 | Hoang et al. 2022 |
| *M. ninhthuanensis* | ZMMU NAP-03780 | Phu Quoc, Kien Giang, Vietnam | MN534571 | Gorin et al. 2020 |
| *M. ninhthuanensis* | KUHE:23856 | Ranong, Thailand | AB598336 | Matsui 2011 |
| *M. daklakensis* | ZISP 14249 | Nam Kar NR, Dak Lak, Vietnam | MT808953 | Hoang et al. 2021 |
| *M. daklakensis* | CIB (VNMN 06858) | Nam Kar NR, Dak Lak, Vietnam | MT808954 | Hoang et al. 2021 |
| *M.* cf. *heymonsi* | IEBR A5072 | Kon Plong , Kon Tum, Vietnam | MT808960 | Hoang et al. 2021 |
| *M.* cf. *heymonsi* | IEBR A5073 | Kon Plong, Kon Tum, Vietnam | MT808961 | Hoang et al. 2021 |
| *M.* cf. *heymonsi* | IEBR A5074 | Kon Plong, Kon Tum, Vietnam | MT808962 | Hoang et al. 2021 |
| *M.* cf. *heymonsi* | IEBR A5075 | Kon Plong, Kon Tum, Vietnam | MT808956 | Hoang et al. 2021 |
| *M.* cf. *heymonsi* | VNMN 04244 | Kon Plong, Kon Tum, Vietnam | MT808957 | Hoang et al. 2021 |
| *M.* cf. *heymonsi* | VNMN 04245 | Kon Plong, Kon Tum, Vietnam | MT808958 | Hoang et al. 2021 |
| *M.* cf. *heymonsi* | VNMN 04450 | Kon Plong, Kon Tum, Vietnam | MT808959 | Hoang et al. 2021 |
| *M.* cf. *heymonsi* | IEBR A.5054 | Kon Plong, Kon Tum, Vietnam | ON724214 | Hoang et al. 2022 |
| *M.* cf. *heymonsi* | IEBR A.5055 | Kon Plong, Kon Tum, Vietnam | ON724213 | Hoang et al. 2022 |
| *M.* cf. *heymonsi* | IEBR A.5056 | Kon Plong, Kon Tum, Vietnam | ON724209 | Hoang et al. 2022 |
| *M.* cf. *heymonsi* | IEBR A.5057 | Vu Quang NP, Ha Tinh, Vietnam | ON724218 | Hoang et al. 2022 |
| *M.* cf. *heymonsi* | IEBR A.5058 | Vu Quang NP, Ha Tinh, Vietnam | ON724217 | Hoang et al. 2022 |
| *M.* cf. *heymonsi* | IEBR A.5059 | Vu Quang NP, Ha Tinh, Vietnam | ON724216 | Hoang et al. 2022 |
| *M.* cf. *heymonsi* | IEBR A.5060 | Vu Quang NP, Ha Tinh, Vietnam | ON724215 | Hoang et al. 2022 |
| *M.* cf. *heymonsi* | IEBR A.5061 | Bach Ma NP, Thua Thien Hue, Vietnam | ON724212 | Hoang et al. 2022 |
| *M.* cf. *heymonsi* | IEBR A.5062 | Bach Ma NP, Thua Thien Hue, Vietnam | ON724211 | Hoang et al. 2022 |
| *M.* cf. *heymonsi* | IEBR A.5063 | Bach Ma NP, Thua Thien Hue, Vietnam | ON724210 | Hoang et al. 2022 |
| *M.* cf. *heymonsi* | IEBR A.5064 | Huu Lien NR, Lang Son, Vietnam | ON745797 | Hoang et al. 2022 |
| *M.* cf. *heymonsi* | IEBR A.5065 | Huu Lien NR, Lang Son, Vietnam | ON745798 | Hoang et al. 2022 |
| *M.* cf. *heymonsi* | IEBR A.5066 | Huu Lien NR, Lang Son, Vietnam | ON745799 | Hoang et al. 2022 |
| *M.* cf. *heymonsi* | IEBR A.5067 | Trang Dinh, Lang Son, Vietnam | ON724208 | Hoang et al. 2022 |
| *M.* cf. *heymonsi* | IEBR A.5068 | Pu Mat NP, Nghe An, Vietnam | ON724219 | Hoang et al. 2022 |
| *M.* cf. *heymonsi* | IEBR A.5069 | Pu Mat NP, Nghe An, Vietnam | ON724220 | Hoang et al. 2022 |
| *M.* cf. *heymonsi* | IEBR A.5070 | Pu Mat NP, Nghe An, Vietnam | ON724221 | Hoang et al. 2022 |
| *M.* cf. *heymonsi* | IEBR A5071 | Pu Mat NP, Nghe An, Vietnam | ON724223 | Hoang et al. 2022 |
| *M.* cf. *heymonsi* | ND.17.16 | Nam Dong, Thanh Hoa, Vietnam | ON724225 | Hoang et al. 2022 |
| *M.* cf. *heymonsi* | ND.17.17 | Nam Dong, Thanh Hoa, Vietnam | ON724224 | Hoang et al. 2022 |
| *M.* cf. *heymonsi* | ND2.17.26 | Nam Dong, Thanh Hoa, Vietnam | ON724223 | Hoang et al. 2022 |
| *M.* cf. *heymonsi* | AMNH A163850 | Vinh Phuc, Vietnam | DQ283382 | Frost et al. 2006 |
| *M.* cf. *heymonsi* | — | Ha Giang, Vietnam | AY458596 | Zhang et al. 2005 |
| *M. heymonsi* | KUHE:50505 | Taiwan, China | LC465686 | Tominaga et al. 2019 |
| *M. pineticola* | VNMN07719 | Bi Duop Nui Ba NP, Lam Dong, Vietnam | MT819964 | Hoang et al. 2020 |
| *M. neglecta* | VNMN07344 | Bi Duop Nui Ba, Lam Dong, Vietnam | MT808928 | Hoang et al. 2020 |
| *M. xodangorum* | IEBR A.4913 | Kon Plong, Kon Tum, Vietnam | ON745755 | Hoang et al. 2022 |
| *M. xodangorum* | IEBR A.4905 | Tam Duong, Lai Chau, Vietnam | ON745737 | Hoang et al. 2022 |
| *M. xodangorum* | IEBR A.4906 | Tam Duong, Lai Chau, Vietnam | ON745738 | Hoang et al. 2022 |
| *M. xodangorum* | IEBR A.4907 | Tam Duong, Lai Chau, Vietnam | ON745739 | Hoang et al. 2022 |
| *M. xodangorum* | IEBR A.4910 | Tam Duong, Lai Chau, Vietnam | ON745740 | Hoang et al. 2022 |
| *M. xodangorum* | IEBR A.4908 | Tam Duong, Lai Chau, Vietnam | ON745741 | Hoang et al. 2022 |
| *M. xodangorum* | IEBR A.4909 | Tam Duong, Lai Chau, Vietnam | ON745742 | Hoang et al. 2022 |
| *M. xodangorum* | IEBR A.4911 | Tam Duong, Lai Chau, Vietnam | ON745743 | Hoang et al. 2022 |
| *M. hmongorum* | KIZ 032771 | Menghan, Mengla, Yunnan, China | OQ726395 | This study |
| *M. hmongorum* | KIZ 032772 | Menghan, Mengla, Yunnan, China | OQ726396 | This study |
| *M. hmongorum* | KIZ 032775 | Menghan, Mengla, Yunnan, China | OQ726397 | This study |
| *M. hmongorum* | KIZ 032776 | Menghan, Mengla, Yunnan, China | OQ726398 | This study |
| *M. hmongorum* | KIZYPX46609 | Panzhihua, Yuanyang, Yunnan, China | OQ726394 | This study |
| *M. hmongorum* | KIZ 027488 | Panzhihua, Yuanyang, Yunnan, China | OQ726393 | This study |
| **Outgroup** |  |  |  |  |
| *M. marmorata* | IEBR A5076 | Kon Plong, Kon Tum, Vietnam | MN453610 | Hoang et al. 2020 |
