# Supplementary material for: First national record of Microhylahmongorum Hoang, Nguyen, Phan, Pham, Ninh, Wang, Jiang, Ziegler and Nguyen, 2022 (Anura, Microhylidae, Microhyla) in China
Source: Biodivers Data J. 2023 Apr 10;11:e103580. doi: 10.3897/BDJ.11.e103580 (PMC10848633; doi:10.3897/BDJ.11.e103580)
Supplement: Supplementary material 2 — Table S2 [file bdj-11-e103580-s002.docx]

**Table 2.** Measurement (in mm) of and proportions of the *Microhyla hmongorum*.

| **Characters** | **KIZ 027488** | **Ratio (-/SVL)** |
| --- | --- | --- |
| Sex | Male |  |
| SVL | 19.9 |  |
| HL | 6.2 | 32.1% |
| HW | 5.8 | 29.1% |
| SL | 2.9 | 14.6% |
| ED | 1.9 | 9.5% |
| NED | 1.3 | 6.5% |
| INS | 1.9 | 9.5% |
| IOS | 2.1 | 10.6% |
| UEW | 1.4 | 7.0% |
| LAL | 3.4 | 17.1 |
| LAHL | 8.2 | 41.2% |
| LAD | 1.5 | 7.5% |
| HAL | 4.8 | 24.1% |
| FEM | 9.5 | 48.7% |
| TL | 10.5 | 52.8% |
| FTL | 10.4 | 52.3% |
